# Retraction Note: The effect of n-3/n-6 polyunsaturated fatty acids on acute reflux esophagitis in rats

**DOI:** 10.1186/s12944-023-01944-7

**Published:** 2023-10-19

**Authors:** Ze-Hao Zhuang, Jing-Jing Xie, Jing-Jing Wei, Du-Peng Tang, Li-Yong Yang

**Affiliations:** 1 Department of Endoscopy, The First Affiliated Hospital of Fujian Medical University, 20 Chazhong Road, 35005 Fuzhou, China; 2 Department of Endocrinology, The First Affiliated Hospital of Fujian Medical University, 35005 Fuzhou, China

**Retraction Note**: ***Lipids in Health and Disease*15, 172 (2016)**

10.1186/s12944-016-0332-2

The authors have retracted this article. After publication, concerns were raised regarding the western blot data in Fig. 4a. The authors have checked the original images and found duplication and labelling issues in the raw data. As the full-length gel images are no longer available, the authors are unable to verify the data and the reliability of the presented results.

All authors agree to this retraction.

